# Positive-Sense RNA Viruses Reprogramming Membrane Contact Sites to Remodel Organelles and Control Cell Fate

**DOI:** 10.3390/v18090966

**Published:** 2026-09-02

**Authors:** Duo Zhang, Liqingyang Wang, Xiaokui Zhang, Fan Yang, Jihong Zhang, Wei Chen

**Affiliations:** 1 Medical School, Kunming University of Science and Technology, Kunming 650050, China; 2 Zhongshan School of Medicine, Sun Yat-sen University Shenzhen Campus, Shenzhen 518107, China

**Keywords:** positive-sense RNA viruses, membrane contact sites (MCSs), replication organelles, organelle-network cascades, cell fate control

## Abstract

Eukaryotic cells rely on a highly coordinated organelle network to maintain metabolism, signaling, stress adaptation and cell survival. Membrane contact sites (MCSs) provide essential platforms for inter-organelle communication. As obligate intracellular parasites, less attention has been given to how viral perturbation of MCSs may reshape the broader organelle communication network. In this review, we focus on positive-sense RNA viruses and virus-induced membrane contact sites (vMCSs), focusing on ER-associated tethering factors, lipid transfer pathways, calcium signaling modules, and replication organelle–host organelle interfaces. Also we discuss how viruses hijack conserved host tethering systems to connect viral replication compartments with lipid transfer machinery. Further, we explore how viruses establish vMCSs that couple replication compartments to host organelles, thereby coordinating cellular homeostasis. Together, these findings suggest that viral remodeling of MCSs should be understood not only as local manipulation of individual organelles but also as a network-level reorganization of cellular homeostasis.

## 1. Introduction

Eukaryotic cells rely on coordinated communication between membrane-bound organelles to maintain metabolism, signaling, and immune response. Among the mechanisms that organize intracellular organelle communication, membrane contact sites (MCSs) have emerged as key platforms central to the integration of the organelle network [1,2]. MCSs are specialized microdomains in the membranes of two distinct organelles are closely apposed, typically separated by a 10–30 nm gap without fusion, permitting non-vesicular exchange of ions and lipids between organelles [3]. From a functional perspective, MCSs represent attractive targets for viruses because they integrate lipid transfer, calcium signaling, metabolic exchange, and stress responses at discrete inter-organelle interfaces. By manipulating these highly connected hubs, viruses can coordinately redirect multiple host processes to create cellular conditions favorable for viral replication [4,5].

As obligate intracellular parasites, viruses extensively exploit host organelles and their communication pathways to support viral replication. Previous studies and reviews have provided important mechanistic insight into how viral proteins target individual organelles or specific MCS-associated factors [4,5]. For example, hepatitis C virus (HCV) NS5A protein hijacks endoplasmic reticulum (ER)–Golgi lipid transfer machinery to promote replication organelle formation [6], whereas other flaviviruses such as dengue virus (DENV) and Zika virus (ZIKV) remodel ER–mitochondria contacts to modulate mitochondrial respiration and apoptotic sensitivity [7]. However, these interactions have largely been considered as discrete events, with less attention given to how local perturbations may reshape the organization and function of the broader organelle network during infection.

In this review, we shift the perspective from isolated virus–organelle interactions toward a dynamic, system-level understanding of viral manipulation. We first summarize the architecture and functional significance of the host organelle network, with particular emphasis on MCSs as sites of inter-organelle coupling. And we then examine how viruses target established MCSs and discuss how viral disruption of MCS-associated pathways reshapes organelle communication and cellular homeostasis. Furthermore, we analyze the potential and challenges associated with developing novel antiviral strategies targeting host organelle communication networks.

## 2. The Architecture and Functional Integration of Organelle Contact Sites

The organelle network provides the structural basis for the spatial coordination of biosynthesis, signaling, and stress responses in eukaryotic cells. Key resources, including lipids, calcium, and metabolic intermediates, are not uniformly distributed throughout the cytoplasm but are generated, stored, and exchanged at defined organelle interfaces. MCSs are central to this organization, enabling rapid organelles communication and integrate metabolic and signaling pathways across the cell [8,9].

The ER forms a continuous and highly branched membrane network that extends throughout the cytoplasm and serves as a major organizing platform for inter-organelle communication. As the principal site of phospholipid synthesis and a major intracellular calcium store, the ER establishes extensive MCSs with mitochondria, the Golgi apparatus, lipid droplets (LDs), lysosomes, and peroxisomes [10]. Within the ER-centered network, LDs emerge from specialized ER subdomains and remain physically and functionally coupled to the ER, which supplies core lipids and regulates LD formation and activity [11]. Through additional contacts with mitochondria, peroxisomes, and lysosomes, LDs participate in lipid storage, redistribution, β-oxidation, membrane biogenesis, and stress tolerance [12]. Thus, rather than acting as an independent central hub, LDs function as an ER-dependent lipid storage and distribution nodes embedded within the broader ER-centered organelle network [13].

**Figure 1:**
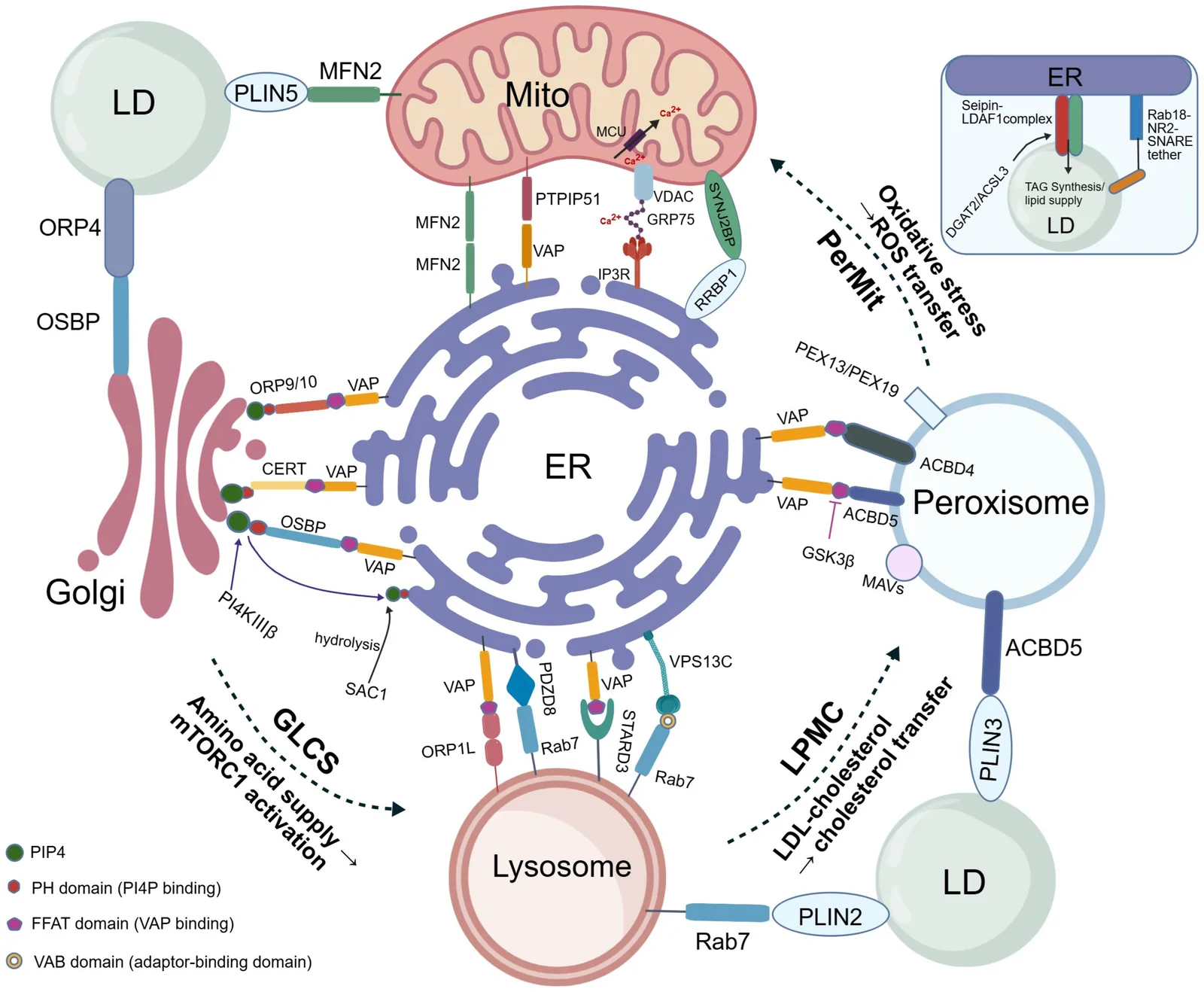
**Architecture of the host organelle network centered on ER-associated MCSs.** The ER is depicted as a continuous, branched membrane network that extends throughout the cytoplasm and forms extensive MCSs with mitochondria, the Golgi apparatus, lipid droplets (LDs), lysosomes, and peroxisomes [1,2]. Within this ER-centered network, LDs function as important lipid storage and distribution nodes, establishing contacts with the ER, mitochondria, lysosomes, and peroxisomes. Key MCS-mediated processes are shown, including calcium transfer from the ER to mitochondria via the IP_3_R-GRP75-VDAC axis, PI4P/cholesterol exchange at ER-Golgi junctions via the VAP-OSBP machinery, and bidirectional lipid flux at ER-LD contacts [14,15,16,17]. VAMP-associated proteins (VAPs) are ER-resident tethering proteins, and their cytosolic major sperm protein (MSP) domains mediate interactions with FFAT-containing partner proteins at opposing membranes. The inset highlights representative molecular components of MCSs, including tethering proteins, lipid transfer proteins, and ion channels, which together provide the structural and functional basis for inter-organelle communication. Created with BioGDP.com [18].

Together, these contacts allow the organelle network to operate as an interconnected system in which perturbation at one contact site can influence multiple downstream processes. This systems-level interdependence is particularly relevant during viral infection. Viruses impose substantial demands on membrane production, energy supply, and cellular stress adaptation, while remodeling organelle interfaces to reshape cellular homeostasis more broadly (Figure 1).

## 3. Mechanisms of Viral Reprogramming of Membrane Contact Sites

The interdependence of the host organelle network creates multiple opportunities for viral intervention at highly connected membrane interfaces. Broadly, viruses exploit MCSs through two related strategies that ultimately reshape inter-organelle communication [5,19]. The first involves hijacking pre-existing host MCS machinery, including tethering factors, lipid transfer proteins, and calcium signaling modules. The second involves the formation of virus-induced membrane contact sites (vMCSs), which connect viral replication compartments with host organelles. The following sections first examine how viruses exploit established host MCS pathways and then consider how they generate contact-like interfaces to support infection.

### 3.1. Hijacking VAP Tethers

VAPA and VAPB are highly conserved ER membrane proteins that function as core tethering factors at many ER-associated MCSs. Their central role depends on interactions with proteins containing a FFAT motif (two phenylalanines in an acidic tract) on opposing membranes, thereby helping to maintain physical coupling between the ER and other organelles [20]. Because FFAT-VAP interactions are widely used across ER contact sites, VAPs have occupied a key position in ER-associated MCSs and are frequent targets of viral subversion [21].

Several viruses exploit this tethering system by encoding proteins that contain FFAT or FFAT-like motifs, allowing them to compete with host FFAT-containing proteins for VAP binding and repurpose VAP-mediated contacts for viral replication. A well-characterized example is HCV, whose non-structural proteins NS5A and NS5B bind directly to VAPs at ER-associated viral replication membranes [22]. These interactions help link the ER to replication organelles (ROs) and create a platform for the recruitment of downstream lipid transfer machinery. A similar principle has been reported in murine norovirus (MNV), in which the NS1/2 protein contains an FFAT motif that interacts with VAP and supports efficient viral replication [23]. Notably, this NS1/2-VAP interaction appears to be conserved in human norovirus (HuNoV), suggesting that viral FFAT mimicry may represent a broader strategy among noroviruses for engaging ER-associated MCS machinery [24].

Taken together, these studies support the idea that VAPs can function as central tethering hubs in virus–host membrane coupling. However, the extent to which different viruses engage shared downstream lipid transfer pathways remains unresolved.

### 3.2. Redirecting Lipid Flux via the OSBP–PI4KIIIβ Axis

Beyond tethering, viruses also exploit lipid transfer pathways at ER-associated MCSs to remodel sterol and phosphoinositide metabolism. A central component of this machinery is oxysterol-binding protein (OSBP), which mediates the non-vesicular counter-exchange of cholesterol and phosphatidylinositol 4-phosphate (PI4P) at ER-Golgi contacts [25,26]. Under physiological conditions, this exchange uses the PI4P gradient to sustain cholesterol transport and maintain the distinct lipid compositions of ER and Golgi membranes. Several positive-sense RNA viruses co-opt this system to generate the lipid-enriched environments required for replication organelle biogenesis.

Poliovirus (PV), coxsackievirus B3 (CVB3), and enterovirus 71 (EV71) encode the non-structural protein 3A, which recruits phosphatidylinositol 4-kinase IIIβ (PI4KIIIβ) to ER-derived replication compartments and promotes local PI4P enrichment [27]. This PI4P-rich environment drives OSBP recruitment and OSBP-mediated cholesterol transfer to viral replication membranes, thereby supporting replication organelle biogenesis. Aichi virus provides a more complete example of this MCS-like lipid transfer module, in which viral proteins coordinate ACBD3, OSBP, VAP-A/B, and SAC1 to couple viral RNA replication sites with an ER-associated cholesterol supply pathway [28]. Related dependence on PI4K- and OSBP-associated lipid remodeling has also been reported for encephalomyocarditis virus (EMCV) and human rhinoviruses (HRVs), suggesting that PI4P-dependent sterol transfer is a broadly used strategy among picornaviruses rather than being restricted to classical enteroviruses [29,30]. Notably, HCV similarly exploits this pathway to promote cholesterol delivery to viral replication membranes. Together, these findings indicate that PI4P-dependent lipid remodeling is a convergent strategy used by different positive-sense RNA viruses.

### 3.3. Exploiting Calcium Signaling at MCSs

Calcium flux at MCSs coordinates bioenergetics, stress responses, and cell survival, making these interfaces attractive targets for viral manipulation [31]. The EV71 2B protein inserts into the ER membrane and promotes depletion of ER calcium stores, thereby activating the ER calcium sensor stromal interaction molecule 1 (STIM1). Activated STIM1 translocate to ER-plasma membrane contacts sites, where it engages Orai1 channels to trigger store-operated calcium entry (SOCE). The resulting increase in cytosolic calcium is partially buffered by mitochondria at ER–mitochondria contact sites, coupling calcium uptake to enhanced tricarboxylic acid cycle activity and ATP production that support viral replication. Disruption of STIM1-Orai1 signaling markedly impairs virus-induced calcium elevation and reduces EV71 replication [32,33].

Other positive-sense RNA viruses also remodel calcium-regulated organelle communication, although not always through canonical MCS machinery. The HCV core protein has been reported to enhance mitochondrial calcium uptake and modulate apoptosis during persistent infection [34]. Porcine reproductive and respiratory syndrome virus (PRRSV) provides a particularly relevant arterivirus example, as infection promotes ER-plasma membrane contact-dependent SOCE and ER-to-mitochondria calcium transfer through IP3R-VDAC1-associated interfaces, thereby linking calcium signaling to autophagy, mitochondrial ROS production, and metabolic remodeling [35,36,37]. Similar principles may extend to flaviviruses such as DENV and ZIKV, which alter ER-mitochondria contact architecture and apoptotic sensitivity [7], as well as to coronaviruses, which perturb endolysosomal calcium-dependent trafficking and lysosome-autophagy pathways [38]. Together, these examples suggest that positive-sense RNA viruses exploit calcium-regulated organelle interfaces to support replication-associated metabolism, trafficking, stress adaptation, and cell fate control.

### 3.4. Inducing vMCSs for Replication

In addition to hijacking pre-existing MCSs, some positive-sense RNA viruses generate virus-induced membrane interfaces that connect their replication compartments with host organelles [4,19].Canonical MCSs are pre-existing host–host organelle interfaces maintained by endogenous tethering and lipid- or ion-transfer machinery [1,3]. In contrast, vMCSs are infection-induced or infection-stabilized interfaces involving a viral replication compartment or virus-remodeled membrane and a host organelle [19]. Because membrane proximity alone does not necessarily indicate functional coupling, we consider evidence of organized membrane apposition together with processes such as lipid transfer, metabolite exchange, or replication-associated trafficking as stronger support for defining an interface as a vMCS.

ROs are virus-induced membrane compartments that support viral RNA synthesis and may include double-membrane vesicles (DMVs), spherules, and other replication-associated structures [39]. Replication complexes (RCs) refer to the functional replication machinery, including viral proteins, viral RNA, and associated host factors, that is typically assembled within or on ROs. Thus, ROs primarily denote the membrane compartment, whereas RCs denote the replication machinery. In contrast, vMCSs refer to contact interfaces linking viral replication structures or virus-remodeled membranes with host membrane systems [19,40].

The PV non-structural protein 2C has been proposed to help establish and maintain interfaces between viral RCs and cellular LDs. These contacts facilitate the transfer of fatty acids from LDs to viral replication sites, where they can support membrane synthesis and RC expansion [41,42,43]. Disruption of 2C-dependent tethering or pharmacological inhibition of LD lipolysis impairs RC formation and reduces viral replication, indicating that RC-LD interfaces support efficient enterovirus replication.

Coronaviruses provides a more complex set of examples. During SARS-CoV-2 infection, ORF3a has recently been shown to recruit LDs to ROs, establishing LD–RO membrane contact sites and promoting microlipophagy to provide lipids required for RO biogenesis [44,45]. The pore complex formed by SARS-CoV-2 NSP3 and NSP4 across the DMV membrane is best understood as a specialized virus-induced membrane interface that may enable RNA export while limiting exposure of replication intermediates to cytosolic innate immune sensors [46,47]. By contrast, NSP6-mediated ER remodeling and ORF3a-associated ER-lysosome perturbation should be interpreted as broader forms of virus-induced remodeling at organelle boundaries rather than as unequivocal vMCSs [48].

**Figure 2:**
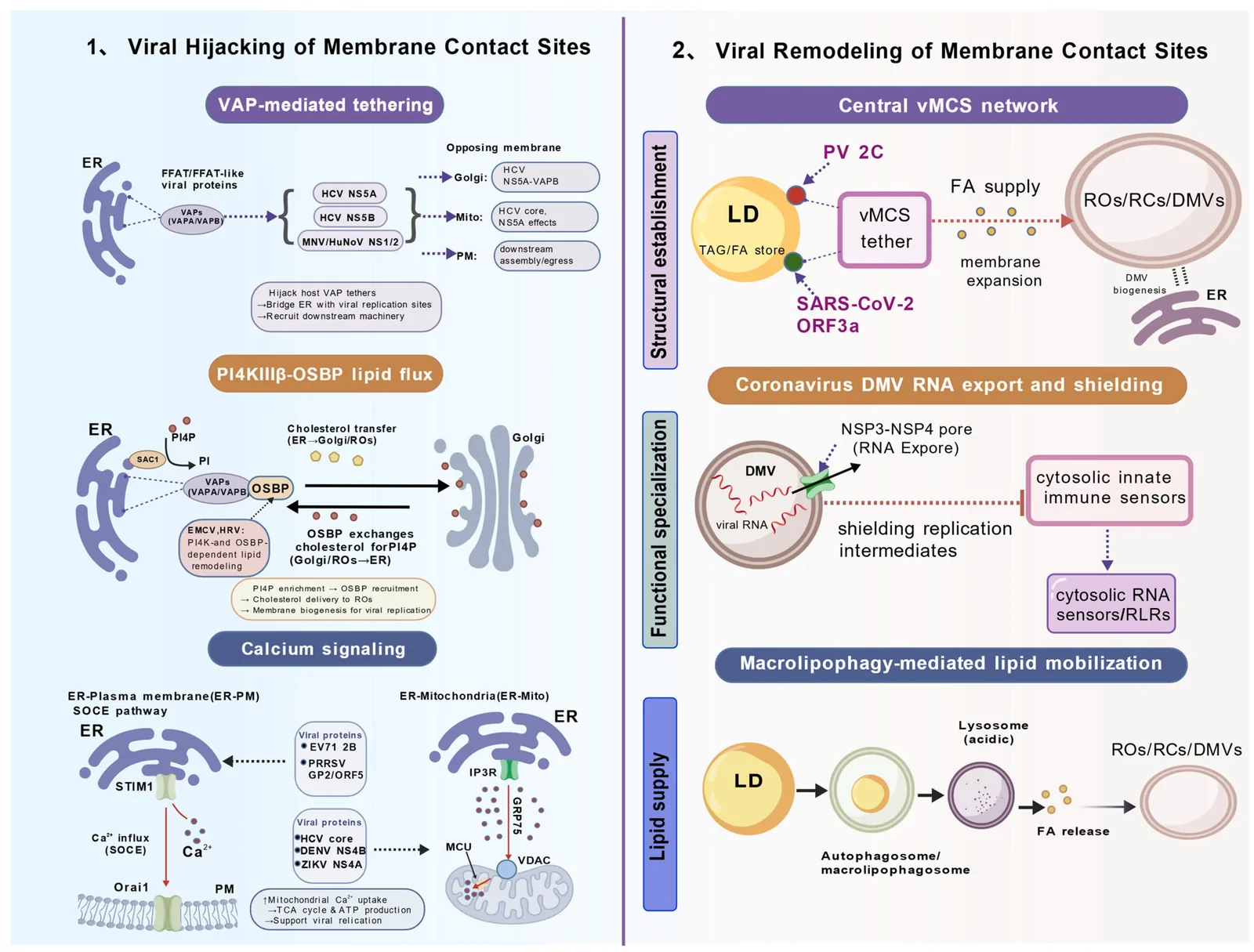
**Viral hijacking and remodeling of membrane contact sites during positive-sense RNA virus infection.** Positive-sense RNA viruses manipulate host MCSs through two major strategies. (1) Viral hijacking of membrane contact sites: viral proteins exploit pre-existing MCS machinery, including VAP-mediated ER tethering, PI4KIIIβ–OSBP-dependent lipid exchange, and calcium signaling at ER–plasma membrane and ER–mitochondria interfaces, thereby supporting lipid redistribution, membrane remodeling, and metabolic adaptation [5,19]. (2) Viral remodeling of membrane contact sites: vMCSs connect replication organelles with host organelles. Representative examples include PV 2C- and SARS-CoV-2 ORF3a-associated LD contacts that promote fatty acid supply and membrane expansion, the SARS-CoV-2 NSP3–NSP4 pore that facilitates viral RNA export and shielding of replication intermediates, and macrolipophagy-mediated lipid mobilization that supports replication-organelle biogenesis. Created with BioGDP.com [18]. Solid arrows indicate directional transport/trafficking; dashed arrows indicate regulatory or functional relationships. Colors are used to distinguish functional modules and molecular components.

Other positive-sense RNA viruses further illustrate the structural diversity of replication-associated interfaces. HCV induces ER-derived membranous webs that are closely associated with LDs, thereby spatially coupling RNA replication with virion assembly [49,50]. Alphaviruses such as chikungunya virus (CHIKV) form spherule-like replication structures at plasma membrane or endolysosomal membranes, providing another example of virus-induced membrane remodeling that connects replication compartments with host membrane systems [51]. Taken together, these observations suggest that virus-induced membrane interfaces expand the range of organelle contacts available to infected cells and allow replication compartments to be coupled more directly to host pathways involved in lipid mobilization, RNA trafficking, and stress adaptation (Figure 2).

## 4. Mechanisms of the Transition from Local Perturbation to Network Remodeling

Viruses perturb MCSs locally by hijacking pre-existing host interfaces or by generating vMCSs. These local changes can extend beyond individual contact sites and drive broader reorganization of the host organelle network, thereby altering communication across multiple organelles and influencing cellular homeostasis. In this section, we examine how local perturbations may propagate through two principal routes: physical propagation through changes in membrane organization and contact-site connectivity and functional propagation through calcium signaling and metabolic coupling. We further consider how these disturbances may be amplified or redirected by viral factors and modulated by host compensatory responses.

### 4.1. Physical Propagation Through MCS Network Interconnectivity

Physical propagation refers to the spread of a local perturbation through changes in membrane architecture, contact-site connectivity, organelle positioning, tether distribution, or lipid organization. In this framework, the defining feature is that structural alteration of one membrane interface changes the physical context of other organelle contacts, rather than the effect being transmitted primarily by diffusible signaling molecules or metabolic intermediates.

Positive-sense RNA viruses provide relevant examples of this form of structural propagation. Flaviviruses such as DENV and ZIKV induce ER-derived replication compartments while remodeling ER-mitochondria contact architecture and mitochondrial organization, suggesting that changes initiated at viral replication membranes can spread to neighboring ER-associated interfaces [7,52]. During SARS-CoV-2 infection, virus-induced replication organelles can become spatially coupled with lipid droplets or endolysosomal membranes, thereby redirecting lipid supply and membrane remodeling toward replication organelle expansion [5,44,53,54]. Although these structures should not all be interpreted as canonical MCSs, they show how local virus-induced membrane remodeling can reorganize the spatial relationships among the ER, lipid droplets, mitochondria, and endolysosomal compartments.

Together, these observations suggest that positive-sense RNA virus-induced perturbation of one ER-linked interface can reshape the structural context of other organelle contacts, including ER–mitochondria, ER-LD, and ER–endolysosomal connections, thereby initiating broader functional cascade effects.

### 4.2. Functional Propagation Through Calcium Signaling and Metabolic Coupling

Functional propagation refers to the spread of a local perturbation through signaling, metabolic, or quality-control pathways that are shared by multiple organelles. Unlike physical propagation, it does not require persistent structural remodeling at every downstream contact site; rather, a change initiated at one interface can influence distant organelles through altered Ca^2+^ flux, ATP production, redox state, nutrient signaling, or autophagy-related pathways. [1,55].

Calcium signaling provides one of the clearest routes for such propagation. Intracellular calcium homeostasis is coordinated across the ER, mitochondria, lysosomes, endosomes, and the plasma membrane, with MCSs spatially organizing calcium release, uptake, and replenishment [33,56]. ER–mitochondria contacts are central to this network, while ER–lysosome and ER–plasma membrane interfaces also contribute by shaping local calcium transfer and store-operated calcium entry [56,57]. Lysosomal calcium channels are particularly important because they couple lysosomal calcium handling to autophagy, nutrient sensing, and membrane trafficking. Consistent with this, Middle East respiratory syndrome coronavirus (MERS-CoV) pseudovirus translocation depends on NAADP-sensitive endolysosomal TPCs, including TPC1 and TPC2. Since TPC1 regulates Ca^2+^ flux at ER–endosome contacts, its disruption may propagate calcium-dependent effects across the organelle network [58,59,60,61]. Disruption at one site may affect calcium-dependent functions in multiple organelles and trigger a broader functional cascade.

Metabolic coupling provides a second route for propagation and amplification. Altered ER–mitochondria calcium transfer can reshape mitochondrial respiration and ATP production, thereby influencing AMPK-mTOR signaling, autophagy, and stress adaptation [62,63,64,65]. During flavivirus infection, DENV and ZIKV remodel ER–mitochondria contact architecture and alter mitochondrial respiration and apoptotic sensitivity, illustrating how local changes in organelle coupling can influence broader metabolic states [7,52]. If lysosomal function is simultaneously compromised, as reported during SARS-CoV-2 infection through disruption of autophagic flux and lysosome-associated pathways, organelle quality control may become incomplete [53,66]. The resulting accumulation of damaged mitochondria, undegraded cargo, and reactive oxygen species can further disturb calcium homeostasis and mitochondrial integrity, creating a positive feedback loop that amplifies the initial perturbation. This cascade eventually triggers broader metabolic abnormalities, expanding the functional consequences of local virus-induced organelle remodeling.

### 4.3. Active Modulation of Cascade Trajectory by Viruses

Viruses do not simply trigger organelle network disturbances passively; in many cases, they also modulate the pace and extent of cascade progression to match their replication strategy. In this sense, the trajectory of a virus-induced organelle cascade is not fixed but can be shaped by viral factors in ways that support distinct infection outcomes [5].

Persistent viruses such as HCV illustrate how cascade progression can be restrained to preserve organelle function during prolonged infection. HCV has been reported to alter ER-mitochondria calcium flux at mitochondria-associated membranes (MAMs) in ways that limit apoptotic signaling while maintaining mitochondrial metabolic activity [67,68]. This strategy is further supported by HCV-induced mitochondrial fission and mitophagy, which may help remove damaged mitochondria and delay premature cell death [69,70]. Thus, viral success during persistent infection depends not only on perturbing organelle contacts but also on preventing excessive amplification of organelle dysfunction.

DENV and ZIKV may represent a more finely tuned mode of cascade control. Both viruses remodel ER–mitochondria contact architecture in infected cells, and these structural changes are accompanied by altered abundance of several ER-mitochondria tethering proteins. Current evidence is consistent with the idea that partial reorganization of these interfaces helps maintain a replication-permissive cytoplasmic environment while delaying the onset of apoptosis [7,52]. PRRSV provides a complementary arterivirus example, in which GP5-associated ER–mitochondria calcium transfer promotes mitochondrial reactive oxygen species production and autophagy, thereby redirecting a potentially damaging stress cascade toward a replication-supportive state [36,37]. In this setting, the relevant effect may be neither complete suppression nor rapid amplification of the cascade but rather adjustment of organelle coupling to balance replication efficiency with cell survival.

**Figure 3:**
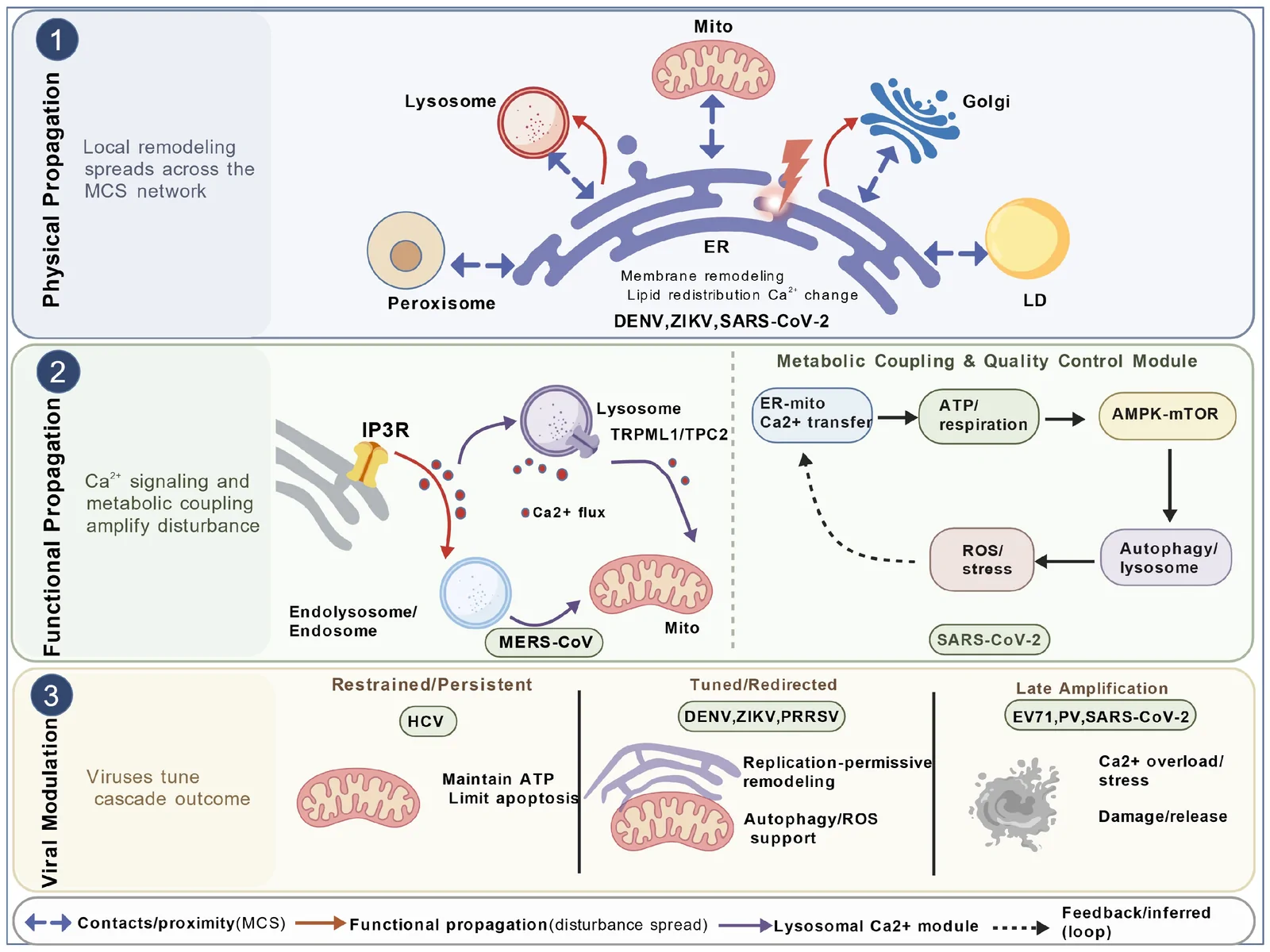
**Conceptual model of virus-induced propagation of MCS perturbations across organelle networks.** Local remodeling of ER-associated membrane contact sites may alter membrane architecture, lipid distribution, and organelle proximity, allowing structural perturbations to extend from one contact site to neighboring ER-linked interfaces [2,4,10]. These changes can be functionally coupled to Ca^2+^ signaling and metabolic communication among the ER, mitochondria, endosomes, and lysosomes. Disrupted Ca^2+^ transfer, mitochondrial respiration, AMPK–mTOR signaling, autophagy–lysosome function, and ROS production may form feedback loops that amplify local disturbances into broader organelle-network dysfunction [10,33,56,73]. Representative positive-sense RNA viruses are shown to illustrate distinct cascade trajectories: HCV may limit excessive mitochondrial damage [68], DENV/ZIKV and PRRSV may redirect organelle coupling toward replication-permissive remodeling [37,52], whereas EV71, PV, and SARS-CoV-2 may be associated with late-stage stress amplification and organelle dysfunction [32,66,72]. Created with BioGDP.com [18].

By contrast, viruses associated with more overtly lytic outcomes may benefit from stronger late-stage amplification of organelle dysfunction. EV71 provides a clear example, as early store-operated calcium entry supports mitochondrial ATP production and replication, whereas later excessive calcium loading promotes mitochondrial damage and apoptosis [32,71]. PV may follow a related trajectory, in which the PI4KIIIβ–OSBP-dependent lipid remodeling described above contributes to ER stress, lysosomal dysfunction, oxidative stress, and cell death at later stages of infection [72]. SARS-CoV-2 infection may represent another route toward cascade amplification, as disruption of autophagic flux can impair organelle quality control and intensify both mitochondrial and inflammatory stress [53,66]. In such cases, viral replication appears to depend not on preventing the cascade altogether but on delaying or redirecting it until release becomes advantageous (Figure 3).

## 5. Network Collapse and Cell Fate Determination

Viral remodeling of membrane contact sites does not remain confined to individual organelle interfaces. By altering lipid transfer, calcium flux, membrane architecture, and organelle quality control, viral proteins can convert local perturbations into broader organelle-network stress. The outcome depends on whether host compensatory pathways can restore network homeostasis or whether persistent perturbation drives irreversible collapse and cell fate transition [74].

### 5.1. Host Compensatory Responses to Organelle Network Stress

Virus-induced perturbation of the organelle network initiates a dynamic interplay between viral manipulation and host compensatory responses. To restore intracellular homeostasis, host cells activate several protective pathways that buffer organelle dysfunction and limit cascade propagation. One major response is the unfolded protein response (UPR), which is activated during infections with viruses such as DENV and coronaviruses [75,76]. Through the IRE1/XBP1, PERK/eIF2α, and ATF6 signaling branches, thereby promoting protein folding, reducing ER load, and supporting membrane repair.

Autophagy provides a second layer of protection by removing damaged organelles. During viral infection, however, this pathway can also be redirected to favor viral replication, as reported for PV and HCV [76], reflecting the dual role of autophagy during infection. Mitochondrial remodeling represents another adaptive response to infection-induced stress. For example, HCV can alter mitochondrial fission and mitophagy, which may facilitate the removal of damaged mitochondria and help preserve cellular metabolic function [69,77]. More generally, mitochondrial fusion and fission are regulated by proteins such as MFN1/2, OPA1, DRP1, and FIS1 [78]. Lysosomal stress responses are also modulated during viral infection. Betacoronaviruses have been shown to alter TFEB and TFE3 activity, linking infection to lysosomal and autophagy-related pathways [79], while TFEB/TFE3-dependent programs normally contribute to lysosomal biogenesis and degradative capacity [80,81].

Together, these responses act not as isolated defenses but as coordinated attempts to stabilize organelle network function during infection. During persistent or high-intensity infection, however, compensatory pathways may become incomplete, exhausted, or maladaptive. Thus, the failure of compensation represents a critical inflection point at which reversible stress responses give way to self-reinforcing organelle network dysfunction.

### 5.2. Cell Fate Outcomes of Organelle Network Dysfunction

Organelle network dysfunction progresses beyond the point of compensation, infected cells may enter distinct fate trajectories depending on the severity, duration, and pattern of damage. Apoptosis is one common outcome and is closely associated with mitochondrial outer membrane permeabilization (MOMP), which integrates upstream signals including calcium overload, ROS accumulation, and ATP depletion [82,83]. Viruses modulate the timing and extent of this process in different ways. Lytic viruses such as EV71 and PV tend to favor late-stage mitochondrial damage and apoptosis, thereby facilitating virion release [32,84]. By contrast, persistent viruses such as HCV dampen apoptotic signaling while preserving metabolic activity, allowing infected cells to remain viable over longer periods [67,85]. DENV and ZIKV may occupy an intermediate position, as remodeling of ER-mitochondria contacts can adjust mitochondrial respiration and apoptotic sensitivity to maintain a replication-permissive state [7,52].

However, not all dysfunctional organelle networks culminate immediately in apoptosis. Senescence may arise under conditions of sublethal but persistent organelle stress, particularly when chronic ER stress, mitochondrial dysfunction, and lysosomal defects remain unresolved [86,87]. In chronic HCV infection, prolonged ER stress, lipid metabolic imbalance, and mitochondrial dysfunction may contribute to a compensated but pathological state associated with progressive liver injury [85]. In other settings, inflammatory cell death, including necroptosis and pyroptosis, may emerge when lysosomal failure is coupled to excessive mitochondrial ROS production [88,89]. This is particularly relevant to SARS-CoV-2 infection, in which lysosome-autophagy disruption and mitochondrial stress can amplify inflammatory signaling [90], and to EV71 infection, where late-stage calcium overload and mitochondrial injury promote lytic or apoptotic cell death [32]. Therefore, cell fate is shaped not by damage to a single organelle in isolation but by the integrated pattern of dysfunction across the broader organelle network.

## 6. Therapeutic and Future Perspectives

The evidence discussed in this review supports a system-level view of viral pathogenesis in which viruses remodel the host organelle network rather than simply perturbing individual organelles or MCSs in isolation. Firstly, the ER emerges in many viral systems as a major organizing platform for viral perturbation because of its extensive participation in membrane contact architecture and biosynthetic exchange [91]. Secondly, both pre-existing MCSs and virus-induced membrane interfaces recur as important sites at which viruses redirect lipid transfer, calcium signaling, and membrane remodeling [4]. Thirdly, viruses do not merely trigger organelle network dysfunction passively, but they can shape the timing and extent of cascade progression in ways that are consistent with their broader infection strategies. Finally, mitochondria and lysosomes frequently emerge as major downstream effectors because they integrate metabolic stress, calcium imbalance, degradative failure, and apoptotic or inflammatory signaling into distinct cell fate outcomes. Together, these patterns support the value of an organelle network cascade model as a unifying framework for understanding viral manipulation.

This perspective also highlights several priorities for future research. However, the organelle network cascade model remains largely conceptual, and direct links between specific MCS changes and viral replication remain unclear. A central challenge will be to distinguish bona fide virus-induced membrane contact sites from broader forms of infection-associated membrane remodeling, particularly in systems where ultrastructural and functional evidence remain incomplete. It will also be important to define how local perturbations propagate across the organelle network in a temporally resolved manner and to determine which nodes act as the most influential amplifiers or buffers in different viral infections. Addressing these questions will require integrated approaches that combine live-cell imaging [92], spatial proteomics [93], organelle-resolved metabolomics [94], and targeted perturbation of MCS components [95]. CRISPR-based screening could help identify MCS components that are important for viral replication and clarify how MCS remodeling is linked to innate immune responses and cellular metabolism [96]. More broadly, this framework suggests that antiviral strategies may benefit from targeting not only individual organelles or viral proteins but also the connectivity and adaptive capacity of the organelle network itself.

## Data Availability

The original contributions presented in this study are included in the article. Further inquiries can be directed to the corresponding author.
